# Metabolome in Tibialis and Soleus Muscles in Wild-Type and *Pin1* Knockout Mice through High-Resolution Magic Angle Spinning ^1^H Nuclear Magnetic Resonance Spectroscopy

**DOI:** 10.3390/metabo14050262

**Published:** 2024-05-06

**Authors:** Valeria Righi, Martina Grosso, Renata Battini, Takafumi Uchida, Anna Gambini, Susanna Molinari, Adele Mucci

**Affiliations:** 1 Department of Life Quality Studies, University of Bologna, Campus Rimini, 47921 Rimini, Italy; valeria.righi2@unibo.it; 2 Department of Life Sciences, University of Modena and Reggio Emilia, 41125 Modena, Italy; martina.grosso3@scuola.istruzione.it (M.G.); renata.battini@unimore.it (R.B.); 3 Laboratory of Molecular Enzymology, Department of Molecular Cell Science, Graduate School of Agricultural Science, Tohoku University, Sendai 981-8555, Japan; uchidataka@gmail.com; 4 Department of Chemical and Geological Sciences, University of Modena and Reggio Emilia, 41125 Modena, Italy; anna.gambini@unimore.it

**Keywords:** *Pin1* KO mice, metabolomics, HR-MAS NMR spectroscopy, slow and fast myofibers, skeletal muscle, metabolic profile, biomarkers

## Abstract

Skeletal muscles are heterogenous tissues composed of different myofiber types that can be classified as slow oxidative, fast oxidative, and fast glycolytic which are distinguished on the basis of their contractile and metabolic properties. Improving oxidative metabolism in skeletal muscles can prevent metabolic diseases and plays a protective role against muscle wasting in a number of neuromuscular diseases. Therefore, achieving a detailed understanding of the factors that regulate myofiber metabolic properties might provide new therapeutic opportunities for these diseases. Here, we investigated whether peptidyl-prolyl cis-trans isomerase NIMA-interacting 1 (PIN1) is involved in the control of myofiber metabolic behaviors. Indeed, PIN1 controls glucose and lipid metabolism in a number of tissues, and it is also abundant in adult skeletal muscles; however, its role in the control of energy homeostasis in this tissue is still to be defined. To start clarifying this topic, we compared the metabolome of the tibialis anterior muscle (mainly glycolytic) and soleus muscle (oxidative) in wild-type and *Pin1* knockout mice with High-Resolution Magic Angle Spinning (HR-MAS) NMR on intact tissues. Our analysis reveals a clear demarcation between the metabolomes in the two types of muscles and allows us to decode a signature able to discriminate the glycolytic versus oxidative muscle phenotype. We also detected some changes in *Pin1*-depleted muscles that suggest a role for PIN1 in regulating the metabolic phenotype of skeletal muscles.

## 1. Introduction

Skeletal muscle tissue plays key roles in body movements, respiration, posture, and energy homeostasis. It is composed of bundles of multinucleated cells called myofibers. In mammals, each muscle is composed of a heterogenous blend of different types of myofibers, distinguished on the basis of their contraction speed and metabolic properties in three classes: slow-twitch oxidative type I, fast-twitch oxidative IIa, and fast-twitch glycolytic type IIb/x [1]. Slow-twitch type I myofibers rely mostly on oxidative phosphorylation as a source of energy production and are resistant to fatigue. Fast-twitch IIb/x myofibers are glycolytic; they contract more quickly but are more susceptible to fatigue. Compared to IIb/x purely fast fibers, type IIa fast myofibers have higher oxidative capacity and resistance to fatigue. The composition of the body muscles in terms of fast and slow fibers varies because of their function. The postural or antigravity muscles (i.e., soleus, SO) have a higher percentage of slow fibers, while those involved in fast and strong maximal contractions (i.e., tibialis anterior, TA) are richer in fast-twitch fibers. Fiber diversity arises during embryonic development, but the myofiber phenotype can change under the influence of internal and external stimuli such as nutrition, hormones, and workload, a property that is called muscle plasticity [1,2,3]. There is wide interest in identifying the molecular mechanisms that underlie muscle plasticity for the potential therapeutic implications that could arise from this knowledge. As there is a positive link between the proportion of slow-twitch type I myofibers and muscle sensitivity to insulin both in rodents and humans, any treatment that induces a fast to slow fiber shift could have a favorable outcome in the treatment of type 2 diabetes [4,5]. Furthermore, the different types of myofibers exhibit a diverse susceptibility to neuromuscular pathologies, including Duchenne muscular dystrophy, age-related muscle atrophy (i.e., sarcopenia), and disuse atrophy, among others [6].

A potential candidate molecule that could play a role in muscle plasticity is the peptidyl-prolyl cis-trans isomerase NIMA-interacting 1 (PIN1) enzyme. PIN1 catalyzes the cis-trans isomerization of phosphorylated serine or threonine preceding proline (the pSer/Thr-Pro motif) in target proteins, leading to their conformational and functional change. Due to its enzymatic activity, PIN1 controls signaling cascades that operate through proline-directed phosphorylation, including the majority of the pathways that govern muscle plasticity [1]. PIN1 is ubiquitous, and its function is important in several cellular and biological processes, encompassing the regulation of energy homeostasis. A high nutritional state induces *Pin1* gene expression; in turn, *Pin1* gene product promotes fuel storage by triggering the insulin signaling pathway and repressing the activity of AMP-activated protein kinase (AMPK) [7,8,9,10,11,12].

In cancer cells, *Pin1* gene is overexpressed and contributes substantially to the Warburg effect by enhancing glycolysis and suppressing oxidative phosphorylation [13]. Furthermore, PIN1 promotes the generation of fatty acids which are used as energy fuel, membrane synthesis, and cell signaling in rapidly proliferating cancer cells [14]. In skeletal muscles, PIN1 is abundant in muscle progenitors, where it modulates terminal differentiation and fusion to generate postmitotic myofibers [15,16,17,18].

PIN1 is also present in fully differentiated adult muscles, regardless of their slow and fast myofibers content. *Pin1* depletion correlates with a lower level of AMPK phosphorylation under fasting conditions and with the increased expression of mitochondria-related genes in skeletal muscles [12]. Yet, the role played by PIN1 in regulating the metabolic properties of myofibers is still obscure.

Here, we sought to investigate the consequences of *Pin1* depletion on adult muscle metabolic behavior by analyzing the metabolomes of the muscle tissue isolated from adult wild-type (WT) and *Pin1* knockout (KO) mice [19]. To start understanding the role of PIN1 in muscle plasticity, we characterized the metabolic properties of *Pin1*-depleted skeletal myofibers.

In recent years, omics-based approaches have shown great potential to highlight metabolic mechanisms related to biological processes and various diseases. The metabolome constitutes the set of small metabolites, including substrates, intermediates, and end products, of cellular metabolism, which can immediately reflect the ongoing changes in cellular physiology and the abnormal level or ratio of metabolites that can induce disease [20]. Metabolomics based on nuclear magnetic resonance (NMR) analysis is a non-destructive, robust, and reproducible technique that requires simple sample preparation. NMR spectroscopy has been used to obtain and quantify metabolites in a large number of tissues, including skeletal muscles [21]. NMR analysis can provide hints about the fuels that are preferentially used by skeletal myofibers.

Here, we compared the metabolomes of WT and *Pin1*-KO oxidative and glycolytic muscles by ex vivo HR-MAS (High-Resolution Magic Angle Spinning) NMR spectroscopy. The rationale behind this strategy was first to identify an HR-MAS NMR metabolic signature characteristic of oxidative muscles by comparing the NMR spectra of SO and TA muscles from WT animals (SO_WT_ and TA_WT_). Then, the obtained signature was instrumental in assessing whether the metabolic profiles of *Pin1*-depleted SO and TA muscles (SO_KO_ and TA_KO_) exhibit a metabolic shift towards a more oxidative or glycolytic metabolism.

## 2. Materials and Methods

### 2.1. Mice and Sample Collection

Mice were maintained on standard rodent diet under a 12 h light/12 h dark cycle, with food and water ad libitum. The *Pin1* KO mice were on a mixed 129/Sv and C57L/B6 background. Three-month-old mice were used for all the experiments in this study; at this age, no gross muscular defects have been reported [19]. The genotype of each mouse was verified by a polymerase chain reaction at birth, as described previously [19]. SO and TA muscles were harvested from euthanized *Pin1* KO (N = 10) and WT (N = 10) mice. Muscle samples were cleared of blood and connective tissues and immediately frozen in liquid nitrogen; they were stored at −80 °C until the time of analysis.

### 2.2. NMR Spectroscopy Measurements

The experiments were performed on the AVANCE III HD 600 Bruker spectrometer (BrukerBiospin, Rheinstetten, Germany), with a ^1^H, ^13^C, ^31^P HR-MAS probe equipped with a Bruker Cooling Unit, working at 600.13 MHz on ^1^H. Tissue was introduced in a 12 or 50 μL MAS zirconia rotor (4 mm OD) with 10 μL of deuterated water (D_2_O), closed with a cylindrical insert to increase sample homogeneity, and then transferred into the HR-MAS probe and cooled to 5 °C to prevent tissue degradation processes. Samples were spun at 4000 Hz. After the set up (about 20 min), two different types of one-dimensional (1D) proton spectra were acquired using the following methods:

(*i*) A ^1^H HR-MAS NMR water-suppressed spin-echo Carr–Purcell–Meiboom–Gill (CPMG, cpmgpr standard Bruker pulse sequence) with 4 s water presaturation during relaxation delay, 1 ms echo time (τ), and 180 ms total spin–spin relaxation delay (2n τ), 24 kHz spectral width, 106 k data points, 256 scans, 4 dummy scans.

(*ii*) A sequence for diffusion measurements based on stimulated echo and bipolar-gradient pulses (ledbpgp2s1d, standard Bruker pulse sequence), with big delta 200 ms, eddy current delay Te 5 ms, little delta 2 × 2 ms, sine-shaped gradient with 47 G/cm, followed by a 200 µs delay for gradient recovery, 8 kHz spectral width, 32 k data points, 96 scans, and 4 dummy scans. The assignment of the metabolites was checked by 2D NMR experiments [22]. The chemical shift of the metabolites detected and assigned is reported in Table S1.

### 2.3. NMR Data Processing

The CPMG spectra, normalized with respect to the muscle weight, were transformed with 0.5 line broadening and reduced to 32 k data points, phase- and baseline-corrected, calibrated with respect to alanine doublet at 1.48 ppm, and aligned with the MNova 14.0.1 software package [23]; the residual water signal, regions above 9 ppm, below 0.7 ppm, and other regions lacking signals, or presenting spinning side bands, were cut. Signals in the region 8.6–8.4 ppm, where resonances due to aromatic protons of adenosine monophosphate, anserine, and carnosine are found, were not aligned. The variations in the chemical shifts of the anserine and carnosine signals reflect the small differences in the pH of the muscles. Multivariate statistical analysis was carried out with Metaboanalyst 4.0–6.0, a web-based metabolomics data analysis software [24], on the spectra after Pareto scaling. Univariate and multivariate statistical analysis was also carried out with Metaboanalyst 4.0–6.0 [24] on the weight-normalized dataset obtained by deconvolution after auto-scaling. It was chosen to deconvolute the myo-inositol signal at 3.62 only for the SO series and the left half of the free glycerol signal at 3.65 only for the TA series to limit deconvolution errors due to other overlapping signals. The pathway analysis was performed with Metaboanalyst 6.0 [24] on the deconvoluted signals.

## 3. Results

### 3.1. Detailed Baseline Analysis of WT Mice Muscles

The ^1^H HR-MAS NMR spectra were directly obtained on tissue specimens, without any pretreatment or extraction process. The NMR metabolite analyses allowed for the identification of about thirty intermediary metabolites (Table S1) in skeletal muscles.

**Figure 1:**
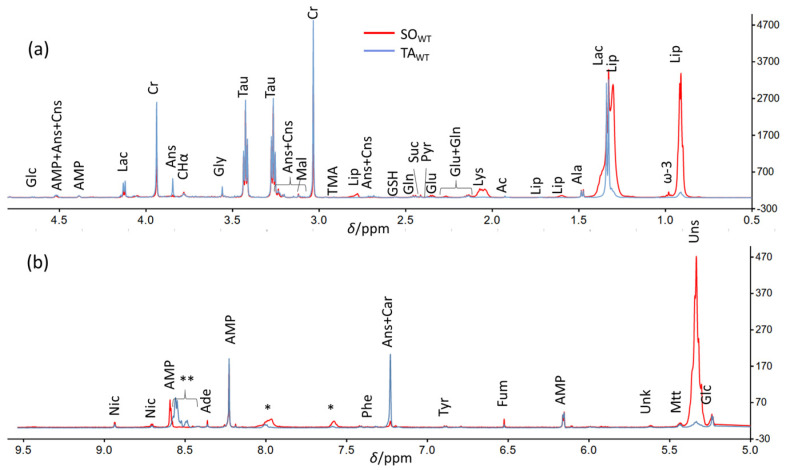
Comparison between the enlarged regions of the HR-MAS ^1^H NMR CPMG mean spectra of the fast-twitch tibialis (blue, TA_wt_) and slow-twitch soleus muscles (red, SO_wt_) in wild-type mouse. (**a**) Low-ppm region; (**b**) high-ppm region. Abbreviations in alphabetical order: acetate (Ac), adenine (Ade), adenosine monophosphate (AMP), alanine (Ala), anserine (Ans), carnosine (Cns), creatine (Cr), fumarate (Fum), glucose (Glc), glutamate (Glu), glutamine (Gln), glutathione (GSH), glycine (Gly), lactate (Lac), lipids (Lip), lysine (Lys), malonate (Mal), maltotriose (Mtt), nicotinamide (Nic), phenylalanine (Phe), pyruvate (Pyr), succinate (Suc), taurine (Tau), trimethylamine (TMA), tyrosine (Tyr), unknown (Unk), unsaturated lipids (Uns). * Spinning side bands. ** In this region of TA_wt_ spectra, Ans, Cns, and AMP signal positions are dependent in terms of pH, and their sum produces a broad feature.

The average CPMG spectra of the two classes, representing the metabolome fingerprint of SO and TA muscles of WT mice (SO_WT_ and TA_WT_, respectively), are reported in Figure 1. The differences between the spectra of the fast-twitch TA and slow-twitch SO muscles can be immediately gathered: SO_WT_ is richer in lipids (chain residuals as broad signals around 0.9, 1.3, 2.1, 2.8, and 5.3 ppm) and fumarate (6.5 ppm) and poorer in lactate (1.32 and 4.12 ppm), anserine (most visible signals around 3.8, 7.2, and 8.5 ppm), and carnosine (most visible signals around 7.2 and 8.5 ppm).

**Figure 2:**
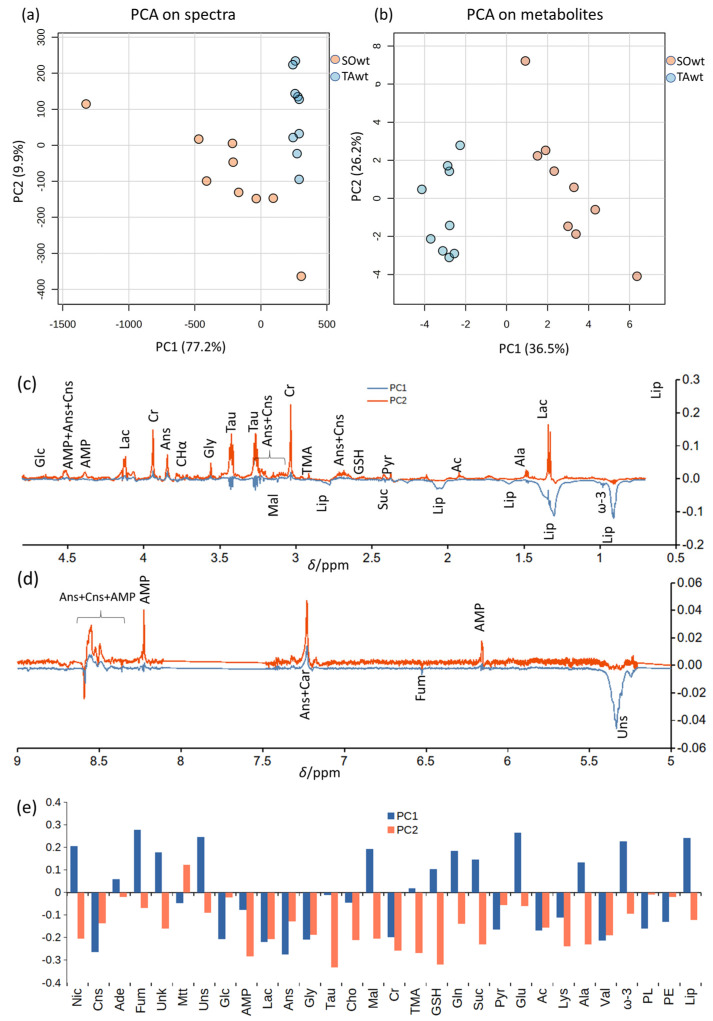
(**a**) Principal component analysis (PCA) on ^1^H CPMG HR-MAS spectra and (**b**) on deconvoluted signals of SO_WT_ (orange) and TA_WT_ (blue) muscles. (**c**,**d**) Enlarged regions of PC1 and PC2 loadings derived from spectra analysis. The region between 7.5 and 8.1 was affected by spinning side bands and was removed. The region around 8.5 ppm was not aligned (see text). (**e**) PC1 and PC2 loadings derived from PCA on deconvoluted signals. Abbreviations in alphabetical order: acetate (Ac), adenine (Ade), adenosine monophosphate (AMP), alanine (Ala), anserine (Ans), carnosine (Cns), choline (Cho), creatine (Cr), fumarate (Fum), glucose (Glc), glutamate (Glu), glutamine (Gln), glutathione (GSH), glycine (Gly), lactate (Lac), lipids (Lip), lysine (Lys), malonate (Mal), maltotriose (Mtt), nicotinamide (Nic), phosphatidylethanolamine (PE), phospholipids (PL), pyruvate (Pyr), succinate (Suc), taurine (Tau), trimethylamine (TMA), tyrosine (Tyr), unknown (Unk), unsaturated lipids (Uns), valine (Val).

To gain further insight into the metabolome of the two different muscles, we applied an explorative unsupervised multivariate analysis (principal component analysis, PCA) to HR-MAS CPMG ^1^H NMR spectra (Figure 2a). The PC1 and PC2 scores plot shows that SO_WT_ and TA_WT_ cluster, as expected, in two well separated regions. Very similar results were obtained through a supervised partial least-squared discriminant analysis (PLS-DA).

The inspection of PC1 and PC2 loading profiles confirms that the separation of PC1 is mainly due to lipids, anserine, carnosine, and fumarate, whereas other small metabolites (lactate, alanine, creatine, taurine, glycine, adenosine monophosphate—AMP) account for PC2 dispersion. TA_WT_ are found, on average, at higher PC1 and PC2 values than SO_WT_. The region 8.4–8.6 ppm also reflects the lack of alignment in the spectra due to differences in pH that shift AMP 8-CH, anserine 2-CH, and carnosine 2-CH signals (Table S1). In particular, negative loadings at 8.59 ppm reflect the position of the 8-CH signals of AMP in SO_WT_.

After the explorative multivariate study, we moved to a quantitative evaluation of the relevant metabolites through the spectral deconvolution of CPMG and diffusion-edited spectra. We tried to deconvolute every metabolite that had one signal not overlapped (or poorly overlapped) to other ones. For instance, in the CPMG spectra, we chose the AMP, anserine, and carnosine signals of 4′-CH, CH_3_, and 2-CH, respectively. To these signals, we added the terminal CH_3_ signal of lipids, a phosphatidylcholine signal at 3.26 ppm, and a signal at 3.0 ppm probably due to phosphatidylethanolamine derived from diffusion-edited experiments (hence, not in the same scale of CPMG signals but, in our opinion, more representative of the less mobile fractions that are underestimated in the CPMG experiments) in order to understand if some changes occur in the lipids pool. The PCA results, based on the deconvoluted metabolite signals common to the SO_WT_ and TA_WT_ classes, are reported in Figure 2b,e and are very similar to those obtained on the spectra.

**Figure 3:**
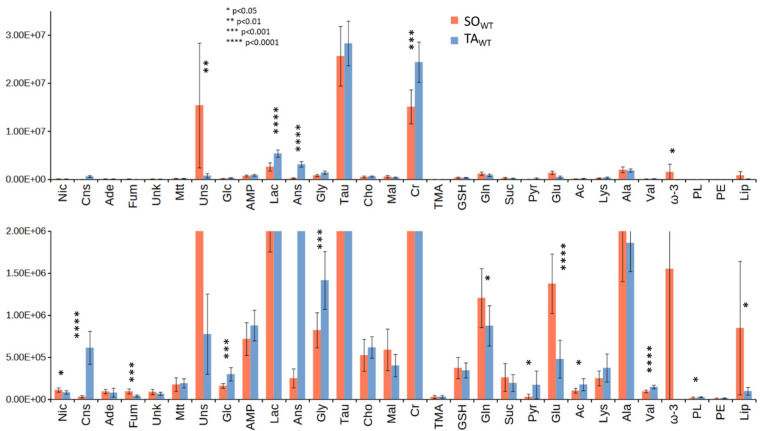
Average amounts (in arbitrary units) of the metabolites derived from signal deconvolution of ^1^H HR-MAS NMR CPMG and diffusion-edited spectra of SO_WT_ (red) and TA_WT_ (blue) classes at different vertical scales. Vertical segments are standard deviations. Asterisks correspond to the raw *p*-values (legend in the figure) obtained by *t*-test. False Discovery Rate (FDR)-corrected *p*-values are reported in Table S2. Abbreviations in alphabetical order: acetate (Ac), adenine (Ade), adenosine monophosphate (AMP), alanine (Ala), anserine (Ans), carnosine (Cns), choline (Cho), creatine (Cr), fumarate (Fum), glucose (Glc), glutamate (Glu), glutamine (Gln), glutathione (GSH), glycine (Gly), lactate (Lac), lipids (Lip), lysine (Lys), malonate (Mal), maltotriose (Mtt), nicotinamide (Nic), phosphatidylethanolamine (PE), phospholipids (PL), pyruvate (Pyr), succinate (Suc), taurine (Tau), trimethylamine (TMA), tyro-sine (Tyr), unknown (Unk), unsaturated lipids (Uns), valine (Val).

A *t*-test on deconvoluted signals was used (Figure 3) to evaluate which metabolites show significant variations between the two groups, finding that not only are the lipids (total lipids, apart from ω-3, related to CH_3_ signal of the acyl chains, unsaturated fraction related to 5.3 ppm signal, labeled Uns, ω-3 fraction, represented by the triplet at 0.98 ppm, labeled ω-3), anserine (3.85 ppm), carnosine (8.5 ppm), lactate (4.12 ppm), and fumarate (6.52 ppm) contents different in SO_WT_ and TA_WT_ but also nicotinamide (8.95 ppm), glucose (4.65 ppm), glycine (3.56 ppm), creatine (3.03 ppm), glutamine (2.45 ppm), pyruvate (2.37 ppm), glutamate (2.35 ppm), acetate (1.92 ppm), and valine (1.05 ppm). The remaining metabolites do not show statistically significant changes.

The fold change (FC) for the deconvoluted signals is reported in Figure S1. From this assay, we can also conclude that SO_WT_ is characterized by higher levels of lipids (unsaturated, ω-3, and total lipids), of nicotinamide, of metabolites associated with the progression of the tricarboxylic acid (TCA) cycle (fumarate, glutamine, glutamate, succinate), and fatty acid synthesis (malonate), and a lower level of glucose, lactate, creatine, pyruvate, acetate, and valine according to the more oxidative metabolism of SO. The histidyl dipeptides, carnosine, and anserine are reduced, as reported in previous studies [25,26].

These results are in line also with those reported for the comparison of SO to masseter and long extensor muscles in young rats [27] for creatine and lactate; they are lower in slow-twitch than in fast-twitch muscles and correlate positively. In mouse, anserine, carnosine, pyruvate, glycine, glucose, acetate, lysine, valine, phospholipids, and phosphatidylethanolamine are clearly lower in slow-twitch than in fast-twitch muscles and correlate positively with creatine as well. The highest correlations of creatine are found for anserine, carnosine, lactate, glycine, and valine. Creatine also correlates positively with taurine and AMP. Some negative correlations were also found to involve carnosine, anserine, glutamate, and fumarate. Eventually, fumarate correlates positively with all lipid signals and glutamate (Figure S2).

Pathway analysis, based on the changes in deconvoluted signals and carried out with Metaboanalyst [24], highlights the principal metabolic routes (see the Section 4, and Table S4) associated with the biomolecules mentioned above.

### 3.2. Comparison between WT and KO Muscles

**Figure 4:**
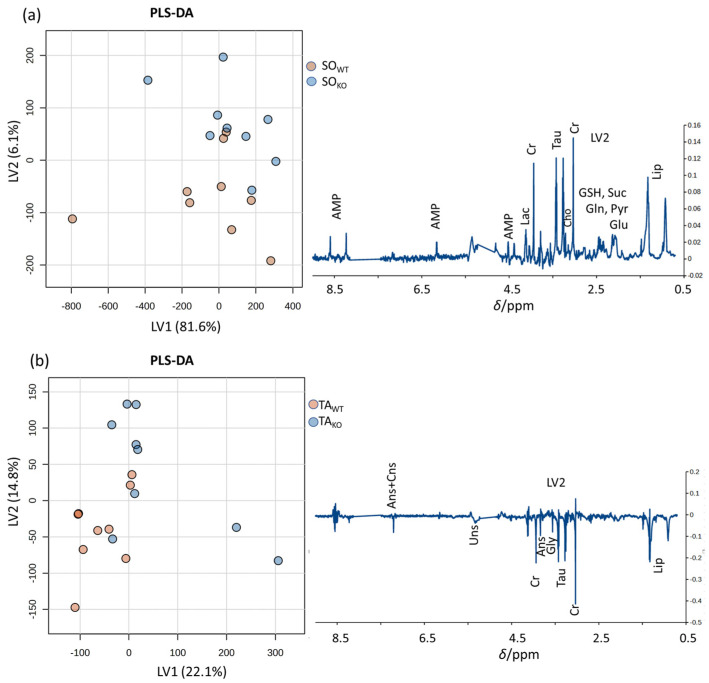
Left: PLS-DA score plots (left), LV2 loading profiles (right) derived from spectra of (**a**) SO_WT_ and SO_KO_, and (**b**) TA_KO_ and TA_WT_ tissue samples highlighting metabolites that could be potentially important for the discrimination between the two classes. Quality parameters of the PLS-DA models are reported in Figure S3. Abbreviations in alphabetical order: adenosine monophosphate (AMP), anserine (Ans), carnosine (Cns), choline (Cho), creatine (Cr), glutamate (Glu), glutamine (Gln), glutathione (GSH), glycine (Gly), lipids (Lip), pyruvate (Pyr), succinate (Suc), taurine (Tau).

The same study approach used for SO_WT_ and TA_WT_ was used to compare SO_KO_ to SO_WT_ and TA_KO_ to TA_WT_. Comparing the spectra of KO muscles with WT muscles, some variations in the metabolic profile are observed both by looking at the mean spectra and by performing multivariate statistical analysis. These differences are captured by the second latent variable (LV2) in PL-SDA analysis and highlight metabolites that might be important for class discrimination: AMP, lactate, creatine, taurine, glutathione, glutamine, succinate, glutamate, and lipids for SO and anserine, carnosine, creatine, taurine, glycine, and lipids for TA (Figure 4). However, they do not appear to be statistically significant.

**Figure 5:**
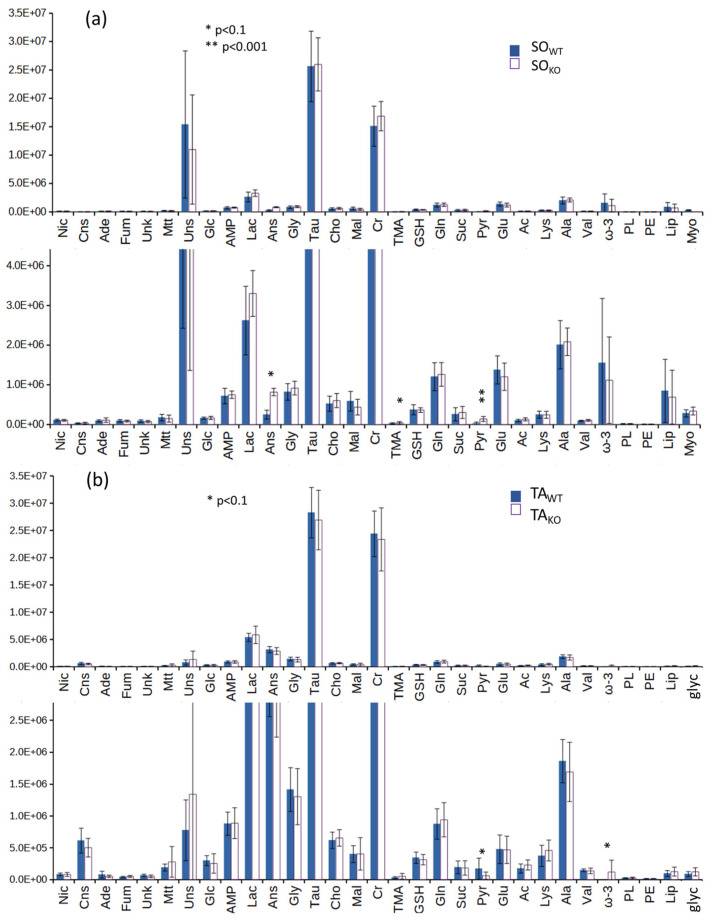
Average amounts (in arbitrary units) of the metabolites derived from signal deconvolution of ^1^H HR-MAS NMR CPMG spectra of (**a**) SO_WT_ (blue) and SO_KO_ (white) (**b**) TA_WT_ (blue) and TA_KO_ (white) at different vertical scales. Vertical segments are standard deviations. Asterisks correspond to the raw *p*-values (legend in the figure) obtained through *t*-test. FDR-corrected *p*-values are reported in Table S2. Abbreviations in alphabetical order: acetate (Ac), adenine (Ade), adenosine monophosphate (AMP), alanine (Ala), anserine (Ans), carnosine (Cns), choline (Cho), creatine (Cr), fumarate (Fum), glucose (Glc), glutamate (Glu), glutamine (Gln), glutathione (GSH), glycine (Gly),free glycerol (glyc), lactate (Lac), lipids (Lip), lysine (Lys), malonate (Mal), maltotriose (Mtt), myo-inositol (Myo), nicotinamide (Nic), phenylalanine (Phe), phosphatidylethanolamine (PE), phospholipids (PL), pyruvate (Pyr), succinate (Suc), taurine (Tau), trimethylamine (TMA), tyro-sine (Tyr), unknown (Unk), unsaturated lipids (Uns), valine (Val).

The univariate analysis of deconvoluted signals (Figure 5) shows that only pyruvate is found to be statistically higher in SO_WT_ at the *p* < 0.001 level, anserine at the *p* < 0.06 level, and trimethylamine at the *p* < 0.08 level. In the case of TA_KO_ and TA_WT_ classes, the differences are even lower: only pyruvate (*p* = 0.080) and ω-3 (*p* = 0.070) are found with *p* < 0.1 after the *t*-test. We also deconvoluted the myo-inositol signal at 3.62 only for the SO series and the glycerol signal at 3.65 only for the TA series, but even these two metabolites do not show appreciable differences between WT and KO classes.

Fold change analysis (Figure S4) shows the opposite behavior of pyruvate, maltotriose, a glycogen intermediate, and unsaturated lipid levels in SO and TA when comparing WT to KO subjects, whereas TMA is enhanced in both KO muscles.

**Figure 6:**
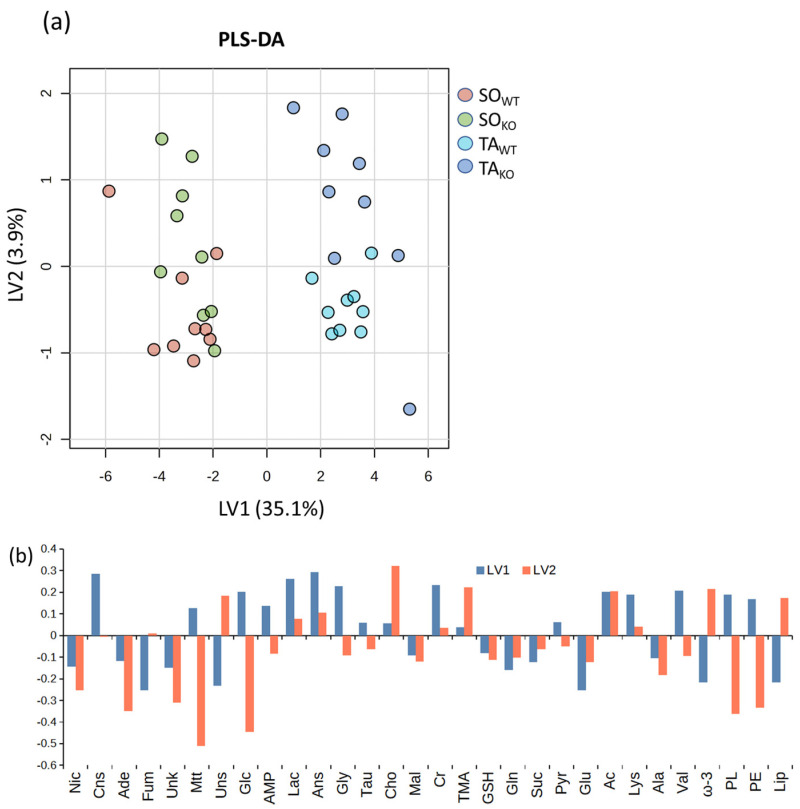
Partial least-square discriminant analysis (PLS-DA) on deconvoluted spectral signals of the four KO and WT classes: (**a**) LV1 and LV2 score plots and (**b**) loading profiles. Quality parameters of the PLS-DA models are reported in Figure S5. Post hoc test for parametric ANOVA is reported in Table S3. Abbreviations in alphabetical order: acetate (Ac), adenine (Ade), adenosine monophosphate (AMP), alanine (Ala), anserine (Ans), carnosine (Cns), choline (Cho), creatine (Cr), fumarate (Fum), glucose (Glc), glutamate (Glu), glutamine (Gln), glutathione (GSH), glycine (Gly), lactate (Lac), lipids (Lip), lysine (Lys), malonate (Mal), maltotriose (Mtt), nicotinamide (Nic), phenylalanine (Phe), phosphatidylethanolamine (PE), phospholipids (PL), pyruvate (Pyr), succinate (Suc), taurine (Tau), trimethylamine (TMA), tyro-sine (Tyr), unknown (Unk), unsaturated lipids (Uns), valine (Val).

When applying multivariate analysis to deconvoluted signals (Figure 6), we observe a clear separation between SO and TA samples, both in WT and KO classes, along the first latent variable in PLS-DA (LV1). The separation of LV1 is mainly due to lipids, some amino acids (valine, glycine, lysine, glutamate), osmolytes (mainly creatine, anserine, and carnosine), and some organic acids (lactate, acetate, and fumarate), and the metabolic picture closely parallels that shown in Figure 2c. Furthermore, PLS-DA shows that the KO samples are shifted in the positive direction of LV2 compared with the WT samples, both within the SO and TA groups. This indicates a corresponding trend of increased signals of lipids, ω-3, and unsaturated lipids and a general decrease in the signals of other metabolites, except choline, acetate, and trimethylamine in KO compared to WT muscles.

Pathway analysis, based on the changes in deconvoluted signals, carried out with Metaboanalyst [24], points to few metabolic routes (see Figure S6 and Table S5) associated with the observed changes in the metabolome only going from SO_WT_ to SO_KO_ muscles.

## 4. Discussion

**Figure 7:**
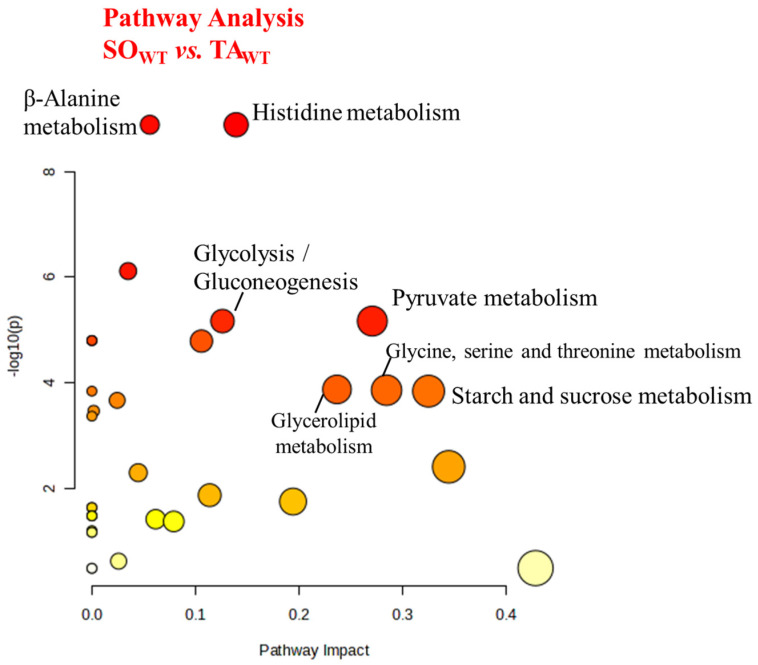
Pathway analysis showing the comparison between SO_WT_ and TA_WT_ metabolism. *p*-values are reported in Table S4.

Our present results indicate that HR-MAS NMR analysis represents a reliable and straightforward method to evaluate the fiber type distribution of isolated mouse skeletal muscles. Consequently, this technique might also be a valid tool to evaluate the efficacity of drug treatments, diet, or physical activity to counteract the altered muscle fiber composition that characterizes some conditions (i.e., bed rest, space flights) or pathologies (like type 2 diabetes, sarcopenia, cancer cachexia, and many others). We defined a metabolome signature that may possibly be used to discriminate the glycolytic or oxidative capacity of skeletal muscles. The most interesting metabolites of this signature are related to the glycolysis/gluconeogenesis pathway with TCA intermediates (fumarate, succinate, glutamate, and glutamine) more abundant in SO, while higher contents of creatine, glutamate, lactate, and pyruvate are detected in glycolytic TA muscles (Figure 7).

These results are coherent with the notion that TA consists chiefly of type II myofibers, while SO is predominantly composed of type I myofibers. Type II myofibers obtain ATP mostly from the anaerobic degradation of glycogen and glucose to pyruvate and lactate, whereas type I myofibers rely on TCA, where citrate reacts with acetylCoA, which derives from pyruvate and fatty acids. Similar results were obtained by comparing different types of muscles in rats [27]. Furthermore, we found that nicotinamide, the precursor of the coenzyme NAD, is more abundant in SO than in TA; NAD is a critical cofactor for pyruvate decarboxylation to acetyl CoA for TCA reactions and for mitochondrial biogenesis. On the other hand, the AMP concentration is higher in TA, possibly reflecting the necessity of fast myofibers to trigger the generation of ATP catalyzed by myokinase. In agreement with the previous literature, we also found that carnosine and its methylated analogue anserine are more abundant in fast TA muscles, where they play important roles as pH buffers and antioxidants, as well as counteracting protein glycation [25]. HR-MAS NMR spectra also revealed a higher content of lipids in slow muscles compared to fast muscles, as was shown previously in mammalian skeletal muscles [28]. This result might possibly be related with the characteristic of oxidative myofibers to use preferentially fat as energy fuel, while the glycolytic counterpart relies mostly on glycogen. Our results may also reflect the higher density of intracellular lipid droplets that have been observed in type I compared to type II myofibers [29]. In agreement with previous results, we also found that unsaturated lipid quantities increase with the oxidative activity of the muscles [30].

The comparison between SO_KO_ and SO_WT_ muscles does not provide a picture of a clear metabolic shift. The increase in trimethylamine (i.e., carnitine) might be related to higher mitochondrial fatty acid oxidation, while the changes observed for other metabolites (pyruvate, acetate, ω-3, anserine, found with a *p* < 0.1) suggest a shift towards a more glycolytic metabolic state. The results obtained by comparing the metabolome of TA_KO_ and TA_WT_ muscles, although only pyruvate and ω-3 were found with a *p* < 0.1, seem to suggest a shift towards a more oxidative metabolic state. In particular, we found a decrease in pyruvate, anserine, and carnosine and an increase in ω-3. Overall, these results allow us to speculate on the role of *Pin1* in the regulation of the muscle metabolic phenotype, with distinct functions in slow and fast muscles. The main changes between WT and KO muscles relate to carbohydrate metabolism, a result we expected considering the important impact of PIN1 on glucose metabolism (see Figure S6 in Supplementary Materials). To define in more detail the phenotype of the *Pin1* KO skeletal muscle, it will be important in the future to perform immunohistochemistry analysis on muscle sections to evaluate whether the observed metabolic variations are accompanied by a shift in the muscle contractile phenotype. High-resolution respirometry will also allow for the assessment of mitochondrial function.

Still, the interpretation of our results is likely troubled by the fact that in our mouse model, the depletion of the *Pin1* gene is ubiquitous and affects the metabolic comportment of the tissues with which muscles establish intense metabolic crosstalk, such as the liver [10]. To demonstrate that the observed metabolic changes are skeletal-muscle-autonomous, it will be necessary to perform metabolomic studies on muscles isolated from muscle-specific conditional KO mice.

Regarding the molecular targets of PIN1 that could mediate the effects of its depletion on muscle metabolism, we can hypothesize that many candidates have to be searched among the various metabolic enzymes that have been shown to be regulated by PIN1 in other cells [11]. For example, it has been shown that in skeletal muscles, PIN1 modulates the function of AMPK, a master regulator of energy homeostasis [12]. In addition to these known molecules, it is conceivable to extend the search of putative PIN1 targets to muscle-specific molecular targets. A potential target candidate may well be the transcription factor myocyte enhancer factor 2 C (MEF2C). Actually, we have previously shown that PIN1 represses the terminal differentiation of muscle progenitors by inhibiting MEF2C function [15]. Notably, in postmitotic muscle cells, MEF2C is involved in the control of myofiber phenotype by favoring slow type I conversion [31]. Based on these premises, it will be important to clarify whether the PIN1-dependent regulation of MEF2C activity could play a role in the changes in the metabolic phenotype of *Pin1* KO muscles.

## 5. Conclusions

Our study shows that a clear metabolic signature, characterizing SO_WT_ and TA_WT_ muscles, can be highlighted by HR-MAS NMR. This technique, applied on isolated mouse skeletal muscles without any pretreatment, represents a comprehensive, reliable, fast, and simple approach to characterize the fiber type composition of mouse skeletal muscles through a combination of metabolites that can be analyzed simultaneously. The comparison of the metabolomes of *Pin1* WT and KO muscles revealed a statistically significant difference for a small number of metabolites. This is not surprising, as we believe that this result is related to the mechanism of action of the PIN1 enzyme in regulating its target signaling pathways. Indeed, PIN1 acts as a molecular timer by modulating, at the same time, an ensemble of signaling molecules generating phenotypes that are always very nuanced. On the basis of its modality of action, it is likely that *Pin1* depletion results in subtle differences spread over a large number of molecules. Nevertheless, we think that PIN1 could play a role in regulating the metabolic phenotype of skeletal muscles and might represent a future target to counteract muscle and metabolic diseases.

## Data Availability

The data presented in this study are available on request from the corresponding authors. The data are not publicly available due to they are still a part of an ongoing study.

## Supplementary Materials

The following supporting information can be downloaded at: https://www.mdpi.com/article/10.3390/metabo14050262/s1, Figure S1: SO_WT_/TA_WT_ fold change; Figure S2: Feature correlation heatmap for SO_WT_ and TA_WT_ classes; Figure S3: SO_KO_/SO_WT_ and TA_KO_/TA_WT_ PLS-DA spectral analyses quality parameters; Figure S4: SO_KO_/SO_WT_ and TA_KO_/TA_WT_ fold change; Figure S5: Quality parameters for PLS-DA on deconvoluted signals of the four classes; Figure S6: Pathway analysis comparing WT and *Pin1* KO metabolism. Table S1: List of ^1^H chemical shifts of the detected metabolites; Table S2: Corrected *p*-values for *t*-test on deconvoluted metabolites signals; Table S3: Post hoc tests for parametric ANOVA on deconvoluted signals of the four classes; Table S4: *p*-values and FDR derived from pathway analysis of SO_WT_ vs. TA_WT_; Table S5: *p*-values and FDR derived from pathway analysis of SO_WT_ vs. SO_KO_; Table S6: *p*-values and FDR derived from pathway analysis of TA_WT_ vs. TA_KO_.
