# Supplementary material for: Metabolome in Tibialis and Soleus Muscles in Wild-Type and Pin1 Knockout Mice through High-Resolution Magic Angle Spinning 1H Nuclear Magnetic Resonance Spectroscopy
Source: Metabolites. 2024 May 6;14(5):262. doi: 10.3390/metabo14050262 (PMC11123315; doi:10.3390/metabo14050262)
Supplement: Supplementary file 1 [file metabolites-14-00262-s001.zip › metabolites-2938146-supplementary.pdf]

Content:

Figure S1. SO_{WT}/TA_{WT} fold change. page 2
Figure S2. Feature correlation heatmap for SO_{WT} and TA_{WT} classes page 2
Figure S3. Quality parameters for SO_{KO} *vs* SO_{WT} and for TA_{KO} *vs* TA_{WT} PLS-DA spectral analyses page 3
Figure S4. SO_{KO}/SO_{WT} and TA_{KO}/TA_{WT} fold change page 4
Figure S5. Quality parameters for SO_{KO}, SO_{WT}, TA_{KO} and TA_{WT} PLS-DA on deconvoluted signals page 4
Figure S6. Pathway analysis comparing WT and *Pin1* KO metabolism. page 5

Table S1. List of ^{1}H and ^{13}C chemical shifts of metabolites in TA and SO muscles pages 6-8
Table S2. P-values from two-tailed t-test on deconvoluted metabolites signals page 9
Table S3. Post-hoc tests for parametric ANOVA on deconvoluted signals of the four classes page 10
Table S4. P-values and FDR derived from pathway analysis of SO_{WT} *vs* TA_{WT} page 11
Table S5. P-values and FDR derived from pathway analysis of SO_{WT} vs SO_{KO} page 12
Table S6. P-values and FDR derived from pathway analysis of TA_{WT} vs TA_{KO} page 13

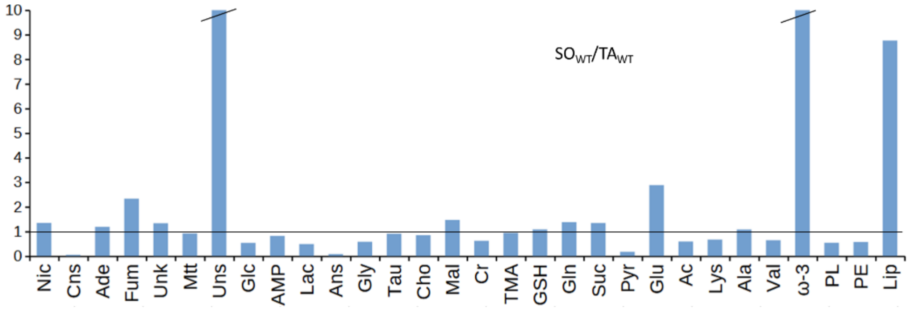

Figure S1. SOWT/TAWT fold change (FC). FC for Uns is about 20 and for ω-3 very high because the triplet at 0.98 ppm in TAWT CPMG spectra is absent.

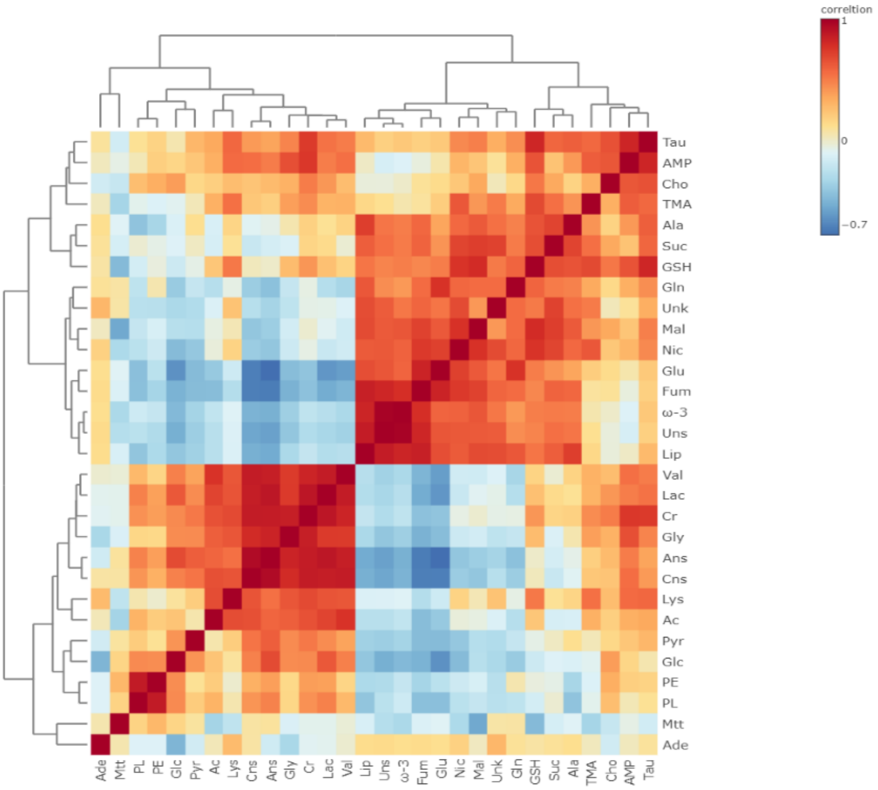

Figure S2: Feature correlation heatmap for SOWT and TAWT classes.

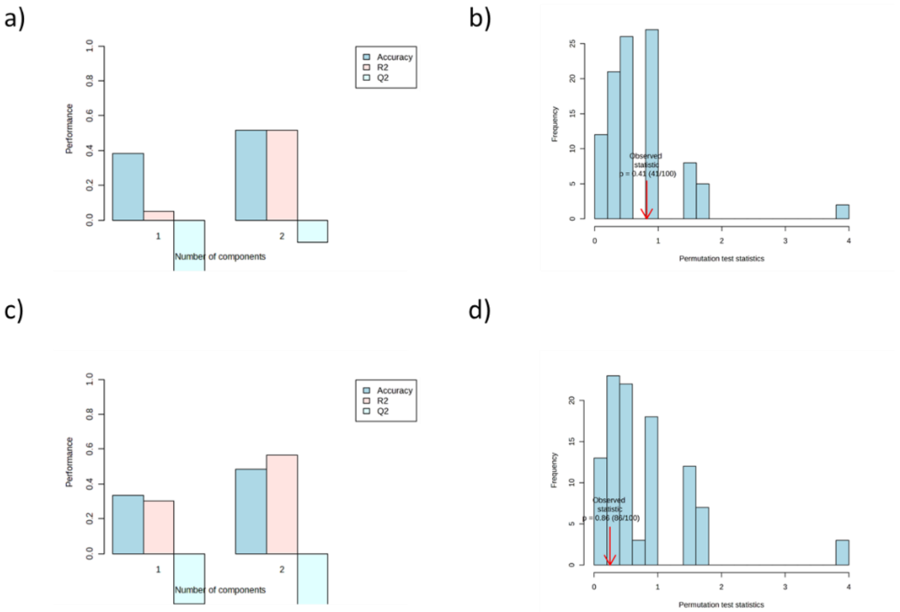

Figure S3. PLS-DA (5-fold) cross validation (a and c) and permutation tests (b and d) for SO_{KO} vs SO_{WT} (a and b) and for TA_{KO} vs TA_{WT} (c and d).

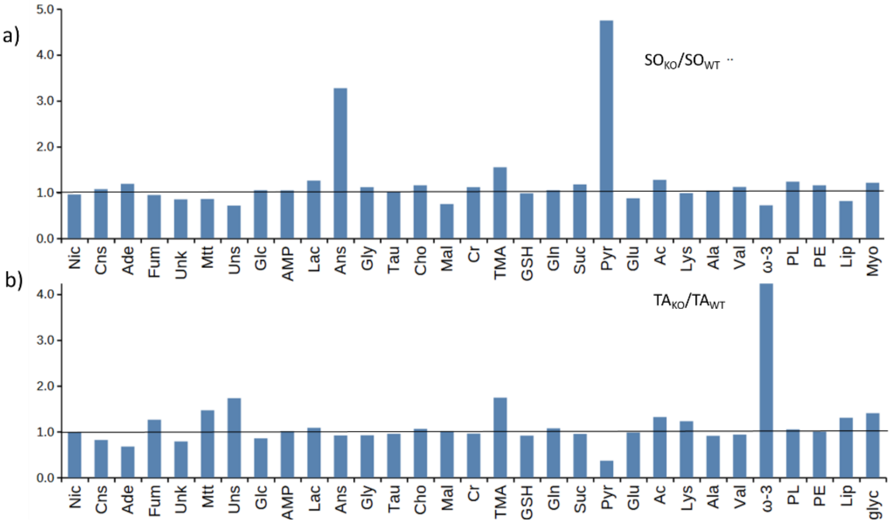

Figure S4. Fold change analysis for a) SO_{KO}/SO_{WT} and b) TA_{KO}/TA_{WT}.

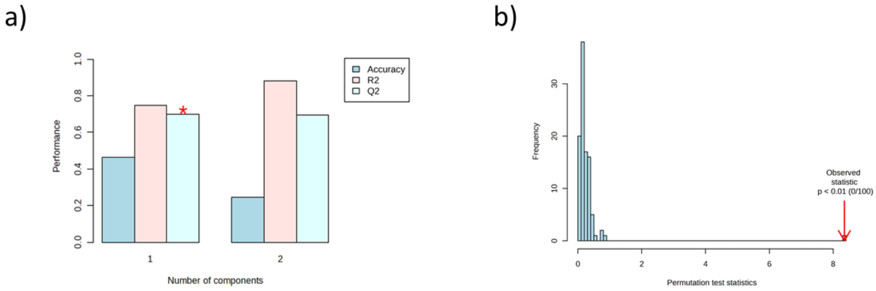

Figure S5. PLS-DA (5-fold) cross validation (a) and permutation test (b) for SO_{KO}, SO_{WT}, TA_{KO} and TA_{WT} classes.

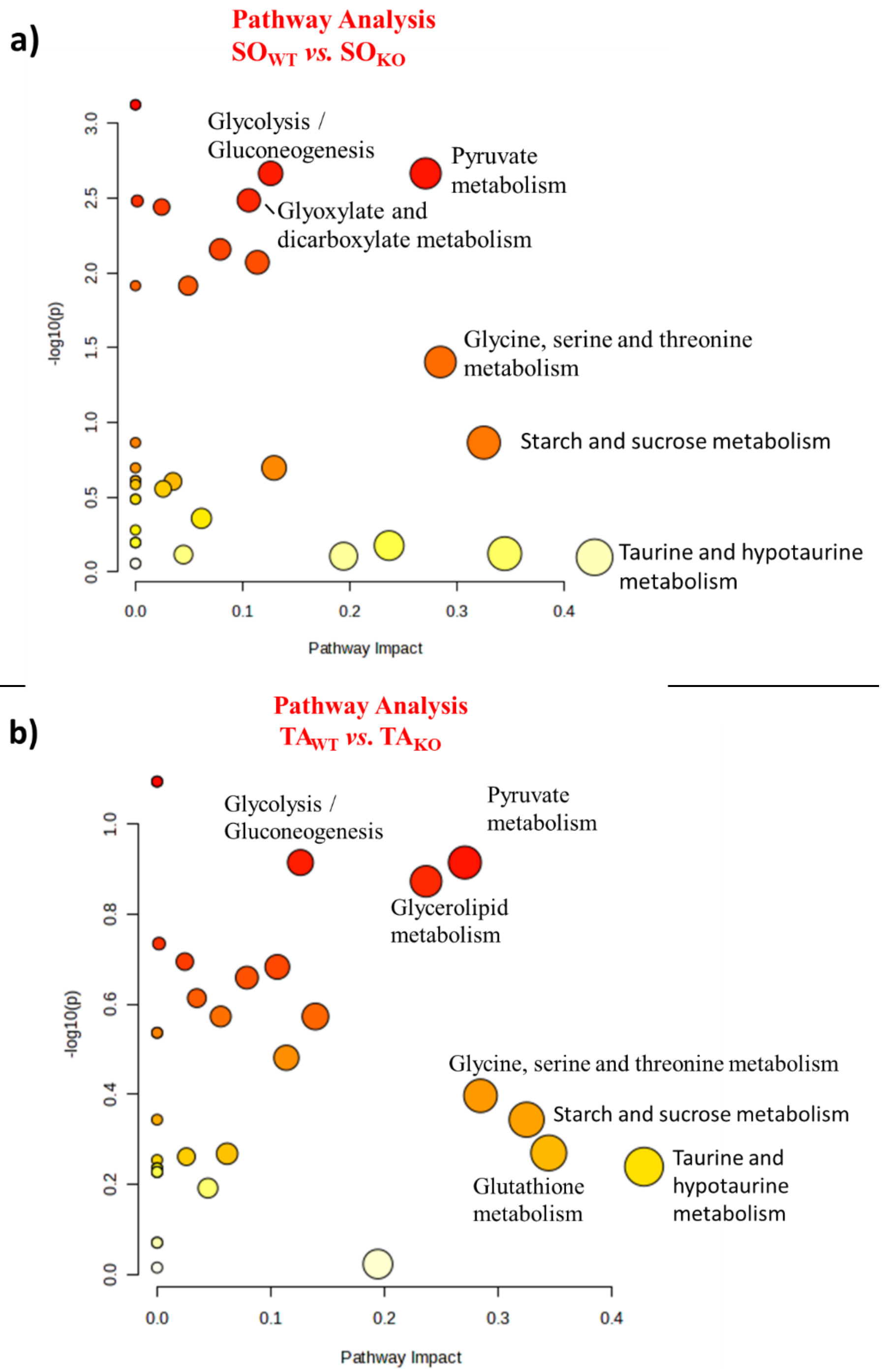

Figure S6. Pathway analysis showing the comparison between WT and *Pin1* KO metabolism in a) SO and b) TA muscles. P-values are reported in Tables S5 and S6.

Table S1. List of ^{1}H and ^{13}C chemical shifts (δ, ppm)[a] of metabolites found in TA and SO muscles.

	Metabolites[b]	δ^1H^a	Assignment	
**1**	fatty acids (Lip)	0.91	CH_3	
	(ω-3)	0.98	CH_3[c]	
		1.37–1.30	$(CH_2)_n$	
		1.60	CH_2-CH_2-C=O	
		2.07–2.04	CH_2CH=CH	
		2.27	CH_2-C=O	
		2.81–2.78	=CH-CH_2-CH=	
	(Uns)	5.30	-CH=CH-	
**2**	leucine	0.96	δ-CH_3	traces
		0.97	δ–CH_3	
		1.71	β–CH_2	
		nd	γ–CH	
		nd	α–CH	
**3**	valine (Val)	0.99 (d)	γ–CH_3	
		1.04 (d)	γ–CH_3	
		2.28	β–CH	
		nd	α-CH	
**4**	lactate (Lac)	1.33 (d)	CH_3	
		4.11 (q)	CH	
**5**	alanine (Ala)	1.48	CH_3	
		3.78	α CH	
**6**	lysine (Lys)	nd	γ–CH_2	
		1.72	δ–CH_2	
		1.90	β–CH_2	
		3.03	ε–CH_2	
		d	α–CH	
**7**	acetate (Ac)	1.92 (s)	CH_3	
**8**	glutamate (Glu)	2.13, 2.07	β–CH_2	
		2.35	γ–CH_2	
		d	α–CH	
**9**	glutamine (Gln)	2.14	β–CH_2	
		2.45	γ–CH_2	
		d	α–CH	
**10**	pyruvate (Pyr)	2.38 (s)	CH_3	
**11**	succinate (Suc)	2.41 (s)	CH_2	
**12**	glutathione (GSH)	2.17	β-CH_{2glu}	
		2.56	γ-CH_{2glu}	
		2.96	β-CH_{2cys}	

		4.58	α-CH_{cys}	
**13**	aspartate	2.70, 2.65	β-CH_2	traces
		nd	α-CH	
**14**	trimethylamine (TMA)	2.90 (s)	CH_3	
**15**	creatine (Cr)	3.03 (s)	CH_3	
		3.94 (s)	CH_2	
**16**	malonate (Mal)	3.12 (s)	CH_2	
**17**	carnosine (Cns)	2.70	$CH_2C{=}O$	
		3.23	CH_2N	
		3.27, 3.09	*CH_2*CH	
		4.51	CH	
		7.23	5-CH	
		8.55-8.44	2-CH	
**18**	anserine (Ans)	2.70	CH_2CO	
		3.23	CH_2N	
		3.24, 3.08	C*H_2*CH	
		3.84	CH_3	
		4.52	CH	
		7.23	5-CH	
		8.58-8.50	2-CH	
**19**	choline (Cho)	3.20 (s)	$N(CH_3)_3$	
		3.50	NCH_2	
		4.06	OCH_2	
**20**	phosphocholine	3.23 (s)	$N(CH_3)_3$	traces
		3.60	NCH_2	
		4.15	OCH_2	
**21**	glycerophosphocholine	3.24 (s)	$N(CH_3)_3$	traces
**22**	phospholipids (PL)	3.26	$N(CH_3)_3$	traces
**23**	taurine (Tau)	3.42 (t)	N-CH_2	
		3.26 (t)	S-CH_2	
**24**	glycine (Gly)	3.56 (s)	CH_2	
**25**	β-glucose (Glc)	3.25	2-CH	
		3.41	4-CH	
		3.47	5-CH	
		3.50	3-CH	
		3.90, 3.73	6-CH_2	
		4.65 (d)	1-CH	
**26**	α-glucose (Glc)	3.42	4-CH	
		3.54	2-CH	
		3.72	3-CH	
		3.83	5-CH	
		3.85, 3.77	6 CH_2	
		5.23 (d)	1-CH	
**27**	maltotriose (Mtt)	3.58	2’-CH and 2”-CH	

		3.72		
		5.44	1’-CH and 1”-CH	
**28**	unknown (Unk)	4.08		
		5.63		
**29**	AMP	4.04	5’-CH_2	
		4.38	4’-CH	
		4.52	3’-CH	
		4.80 (t)	2’-CH	
		6.16 (d)	1’-CH	
		8.23	2-CH	
		8.59 (SO) 8.59-8.53 (TA)	8-CH	
**30**	adenine (Ade)	8.19	2-CH	
		8.36	8-CH	
**31**	glycerol bound in TG	4.31, 4.10	CH_2	
		5.24	CH	
**32**	free glycerol (glyc)	3.65, 3.55	CH_2	
		3.78	CH	
**33**	myo-inositol (Myo)	3.53	1,3-CH	
		4.06	2-CH	
		3.62	4,6-CH	
		3.27	5-CH	
**34**	fumarate (Fum)	6.52	CH	
**35**	tyrosine (Tyr)	6.89	3-CH	traces
		7.18	2-CH	
**36**	phenylalanine (Phe)	7.32	H-*o*	traces
		7.37	H-*p*	
		7.42	H-*m*	
**37**	nicotinamide (Nic)	7.59	5-CH	
		8.23	4-CH	
		8.70 (d)	6-CH	
		8.94	2-CH	

[a] ^{1}H chemical shifts refer to Ala doublet at 1.48 ppm. (s) singlet, (d) doublet, (t) triplet.
[b] abbreviations used in the text are reported in parentheses.
[c] CH_3 from polyunsaturated ω-3 fatty acid chains.
[d] contributes to the peak at 3.78 ppm.

Table S2. Raw and FDR corrected p-values from two-tailed t-test on deconvoluted metabolites signals.

	SOWT *vs* TAWT		SOWT *vs* SOKO		TAWT *vs* TAKO	
	raw p-value	FDR corrected p-value	raw p-value	FDR corrected p-value	raw p-value	FDR corrected p-value
Nic	0.0176	0.0377	0.7840	0.9273	0.9485	0.9890
Cns	0.0000001	2.0E-06	0.8077	0.9273	0.1936	0.6667
Ade	0.4649	0.5364	0.3240	0.8420	0.1891	0.6667
Fum	0.00013	0.0005	0.6655	0.9273	0.1339	0.6667
Unk	0.0919	0.1253	0.5769	0.9273	0.1534	0.6667
Mtt	0.6462	0.6685	0.2715	0.8420	0.2980	0.7324
Uns	0.0038	0.0115	0.7007	0.9273	0.3047	0.7324
Glc	0.00014	0.00054	0.1363	0.8420	0.4531	0.7982
AMP	0.0904	0.1253	0.6303	0.9273	0.9337	0.9890
Lac	0.000002	0.00002	0.3632	0.8662	0.4391	0.7982
Ans	1E-10	2.8E-09	0.0582	0.5579	0.3837	0.7982
Gly	0.00043	0.0014	0.5237	0.9273	0.5570	0.7982
Tau	0.3196	0.3862	0.7972	0.9273	0.5764	0.7982
Cho	0.2360	0.3079	0.2779	0.8420	0.5476	0.7982
Mal	0.0624	0.1040	0.2604	0.8420	0.9655	0.9890
Cr	0.00011	0.00054	0.4848	0.9273	0.6741	0.8707
TMA	0.8266	0.8266	0.0720	0.5579	0.1754	0.6667
GSH	0.5870	0.6289	0.7208	0.9273	0.4702	0.7982
Gln	0.0328	0.0579	0.2453	0.8420	0.5922	0.7982
Suc	0.3219	0.3862	0.3259	0.8420	0.8500	0.9890
Pyr	0.0228	0.0457	0.00075	0.0232	0.0804	0.6667
Glu	0.00001	0.00006	0.7968	0.9273	0.9270	0.9890
Ac	0.0134	0.0309	0.0688	0.5579	0.1371	0.6667
Lys	0.0671	0.1060	0.8766	0.9371	0.2905	0.7324
Ala	0.5320	0.5912	0.7312	0.9273	0.3943	0.7982
Val	0.00002	0.00010	0.6356	0.9273	0.5815	0.7982
ω-3	0.0111	0.0300	0.7778	0.9273	0.0701	0.6667
PL	0.0290	0.0543	0.8636	0.9371	0.7977	0.9890
PE	0.0788	0.1182	0.9146	0.9450	0.9890	0.9890
Lip	0.0120	0.0300	0.9492	0.9492	0.3072	0.7324
Myo	-	-	0.2012	0.8420	-	-
glycl	-	-	-	-	0.1762	0.6667

Table S3. Post-hoc test for parametric ANOVA on deconvoluted signals of the four classes: 0 (SO_{WT}), 1 (SO_{KO}), 2 (TA_{WT}) and 3 (TA_{KO})

	f-value	p-value	-log10(p)	FDR corrected p-value	Fisher's LSD			
Ans	116.94	2.52E-17	16.60	7.55E-16	2 - 0	3 - 0	2 - 1	3 - 1
Cns	56.152	7.57E-13	12.12	1.14E-11	2 - 0	3 - 0	2 - 1	3 - 1
Glu	30.154	1.89E-09	8.72	1.89E-08	0 - 2	0 - 3	1 - 2	1 - 3
Lac	23.496	3.17E-08	7.50	2.37E-07	2 - 0	3 - 0	2 - 1	3 - 1
Fum	17.881	5.37E-07	6.27	3.22E-06	0 - 2	0 - 3	1 - 2	1 - 3
Cr	11.854	2.20E-05	4.66	1.10E-04	2 - 0	3 - 0	2 - 1	3 - 1
Gly	8.2834	3.21E-04	3.49	1.29E-03	2 - 0	3 - 0	2 - 1	3 - 1
Uns	8.1493	3.59E-04	3.45	1.29E-03	0 - 2	0 - 3	1 - 2	1 - 3
Ac	8.0097	4.02E-04	3.40	1.29E-03	2 - 0	3 - 0	3 - 1	
Val	7.9334	4.29E-04	3.37	1.29E-03	2 - 0	3 - 0	2 - 1	3 - 1
Glc	6.7406	1.19E-03	2.93	3.24E-03	2 - 0	3 - 0	2 - 1	3 - 1
ω-3	6.1761	1.96E-03	2.71	4.91E-03	0 - 2	0 - 3	1 - 2	1 - 3
Gln	6.0398	2.22E-03	2.65	5.13E-03	0 - 2	1 - 2	1 - 3	
Lip	5.7664	2.86E-03	2.54	6.12E-03	0 - 2	0 - 3	1 - 2	1 - 3
Lys	5.5371	3.54E-03	2.45	7.08E-03	3 - 0	2 - 1	3 - 1	
Nic	4.563	9.03E-03	2.04	1.69E-02	0 - 2	0 - 3	1 - 2	1 - 3
Pyr	4.1012	1.43E-02	1.84	2.53E-02	1 - 0	2 - 0	2 - 3	
Unk	3.6048	2.38E-02	1.62	3.85E-02	0 - 3	1 - 3		
PL	3.5811	2.44E-02	1.61	3.85E-02	2 - 0	3 - 0	2 - 1	3 - 1
Ade	3.4395	2.83E-02	1.55	4.24E-02	0 - 3	1 - 3		
PE	2.9955	4.52E-02	1.34	6.46E-02	2 - 0	3 - 0	2 - 1	3 - 1
Suc	2.4221	8.40E-02	1.08	1.15E-01	1 - 2	1 - 3		
AMP	1.9305	1.45E-01	0.84	1.88E-01				
Mtt	1.8665	1.55E-01	0.81	1.94E-01	3 - 1			
TMA	1.7419	1.78E-01	0.75	2.14E-01				
Mal	1.5072	2.31E-01	0.64	2.67E-01				
Ala	1.3438	2.78E-01	0.56	3.08E-01				
GSH	1.158	3.41E-01	0.47	3.61E-01				
Cho	1.1366	3.49E-01	0.46	3.61E-01				
Tau	0.4105	7.47E-01	0.13	7.47E-01				

Table S4. P-values and FDR derived from pathway analysis of SO_{WT} *vs* TA_{WT}

	Total Cmpd	Hits	Raw p	-log10(p)	Holm adjust	FDR	Impact
Histidine metabolism	16	3	6.8298e-11	10.166	2.1855e-09	2.1855e-09	0.13934
beta-Alanine metabolism	21	2	1.3139e-09	8.8814	0.00004073	2.1022e-08	0.05597
Porphyrin metabolism	31	2	3.5094e-08	7.4548	1.0528e-06	3.4345e-07	0
Glycolysis / Gluconeogenesis	26	4	4.2932e-08	7.3672	0,001245	3.4345e-07	0.12637
Arginine and proline metabolism	36	3	0.0002104	6.677	5.8911e-06	1.3465e-06	0.02442
Glutathione metabolism	28	3	5.0176e-07	6.2995	1.3548e-05	2.6761e-06	0.36435
Glyoxylate and dicarboxylate metabolism	32	5	6.4209e-07	6.1924	1.6694e-05	2.9353e-06	0.10582
Pyruvate metabolism	23	3	6.7939e-06	5.1679	0.00016985	2.7176e-05	0.27088
Valine, leucine and isoleucine degradation	40	1	1.5834e-05	4.8004	0.00038002	4.6063e-05	0
Valine, leucine and isoleucine biosynthesis	8	1	1.5834e-05	4.8004	0.00038002	4.6063e-05	0
Pantothenate and CoA biosynthesis	20	1	1.5834e-05	4.8004	0.00038002	4.6063e-05	0
Glycerolipid metabolism	16	1	0.0001332	3.8755	0.0027972	0.00031484	0.23676
Galactose metabolism	27	1	0.0001332	3.8755	0.0027972	0.00031484	0
Glycine, serine and threonine metabolism	34	4	0.00013774	3.8609	0.0027972	0.00031484	0.28464
Lipoic acid metabolism	28	2	0.00034154	3.4666	0.0061478	0.00072862	0.0017
Arginine biosynthesis	14	2	0.00044987	3.3469	0.0076478	0.00084681	0.11675
Nitrogen metabolism	6	2	0.00044987	3.3469	0.0076478	0.00084681	0
Butanoate metabolism	15	2	0.00055862	3.2529	0.0083794	0.00099311	0
Alanine, aspartate and glutamate metabolism	28	5	0.0017998	2.7448	0.025198	0.0030313	0.3109
Primary bile acid biosynthesis	46	2	0.005003	2.3008	0.065038	0.0080047	0.04478
Nicotinate and nicotinamide metabolism	15	1	0.017601	1.7545	0.21121	0.02682	0.1943
Cysteine and methionine metabolism	33	1	0.022833	1.6414	0.25116	0.031768	0
Tyrosine metabolism	42	1	0.022833	1.6414	0.25116	0.031768	0
Pyrimidine metabolism	39	1	0.032816	1.4839	0.29535	0.043755	0
Purine metabolism	71	3	0.037946	1.4208	0.30357	0.048571	0.06162
Citrate cycle (TCA cycle)	20	2	0.041809	1.3787	0.30357	0.051457	0.07907
Lysine degradation	30	1	0.067127	1.1731	0.40276	0.076716	0
Biotin metabolism	10	1	0.067127	1.1731	0.40276	0.076716	0
Glycerophospholipid metabolism	36	1	0.23604	0.62702	0.94415	0.26046	0.02582
Taurine and hypotaurine metabolism	8	1	0.31961	0.49538	0.95883	0.33225	0.42857
Propanoate metabolism	22	1	0.32186	0.49233	0.95883	0.33225	0
Selenocompound metabolism	20	1	0.53204	0.27406	0.95883	0.53204	0

Table S5. P-values and FDR derived from pathway analysis of SO_{WT} *vs* SO_{KO}.

	Total Cmpd	Hits	Raw p	-log10(p)	Holm adjust	FDR	Impact
Cysteine and methionine metabolism	33	1	0.00074946	3.1253	0.025482	0.01271	0
Tyrosine metabolism	42	1	0.00074946	3.1253	0.025482	0.01271	0
Glycolysis / Gluconeogenesis	26	4	0.0011214	2.9502	0.035886	0.01271	0.12637
Pyruvate metabolism	23	3	0.0021568	2.6662	0.066859	0.018332	0.27088
Lipoic acid metabolism	28	2	0.003299	2.4816	0.098969	0.022433	0.0017
Citrate cycle (TCA cycle)	20	2	0.0069501	2.158	0.20155	0.039384	0.07907
beta-Alanine metabolism	21	1	0.01216	1.9151	0.34048	0.059063	0
Glyoxylate and dicarboxylate metabolism	32	5	0.02143	1.669	0.57861	0.091077	0.10582
Arginine and proline metabolism	36	3	0.034156	1.4665	0.88804	0.12903	0.02442
Glycine, serine and threonine metabolism	34	4	0.039384	1.4047	0.9846	0.13391	0.28464
Histidine metabolism	16	2	0.045732	1.3398	1	0.14135	0.04918
Alanine, aspartate and glutamate metabolism	28	5	0.065019	1.187	1	0.18422	0.3109
Inositol phosphate metabolism	30	1	0.20119	0.69639	1	0.48861	0.12939
Ascorbate and aldarate metabolism	10	1	0.20119	0.69639	1	0.48861	0
Pyrimidine metabolism	39	1	0.24529	0.61032	1	0.55598	0
Glycerophospholipid metabolism	36	1	0.27787	0.55616	1	0.59047	0.02582
Propanoate metabolism	22	1	0.32593	0.48687	1	0.65187	0
Galactose metabolism	27	2	0.37435	0.42672	1	0.7071	0
Arginine biosynthesis	14	2	0.4313	0.36522	1	0.70865	0.11675
Nitrogen metabolism	6	2	0.4313	0.36522	1	0.70865	0
Purine metabolism	71	3	0.43769	0.35883	1	0.70865	0.06162
Butanoate metabolism	15	2	0.59184	0.22779	1	0.86444	0
Valine, leucine and isoleucine degradation	40	1	0.63562	0.1968	1	0.86444	0
Valine, leucine and isoleucine biosynthesis	8	1	0.63562	0.1968	1	0.86444	0
Pantothenate and CoA biosynthesis	20	1	0.63562	0.1968	1	0.86444	0
Glycerolipid metabolism	16	1	0.66553	0.17683	1	0.8703	0.23676
Selenocompound metabolism	20	1	0.73124	0.13594	1	0.87431	0
Porphyrin metabolism	31	2	0.74814	0.12602	1	0.87431	0
Primary bile acid biosynthesis	46	2	0.7645	0.11662	1	0.87431	0.04478
Nicotinate and nicotinamide metabolism	15	1	0.78404	0.10566	1	0.87431	0.1943
Taurine and hypotaurine metabolism	8	1	0.79717	0.098452	1	0.87431	0.42857
Glutathione metabolism	28	3	0.85275	0.069179	1	0.87662	0.36435
Lysine degradation	30	1	0.87662	0.05719	1	0.87662	0
Biotin metabolism	10	1	0.87662	0.05719	1	0.87662	0

Table S6. P-values and FDR derived from pathway analysis of TA_{WT} *vs* TA_{KO}

	Total Cmpd	Hits	Raw p	-log10(p)	Holm adjust	FDR	Impact
Cysteine and methionine metabolism	33	1	0.080383	1.0948	1	0.78959	0
Tyrosine metabolism	42	1	0.080383	1.0948	1	0.78959	0
Pyruvate metabolism	23	3	0.12163	0.91497	1	0.78959	0.27088
Glycerolipid metabolism	16	1	0.13391	0.8732	1	0.78959	0.23676
Galactose metabolism	27	1	0.13391	0.8732	1	0.78959	0
Glycolysis / Gluconeogenesis	26	4	0.16507	0.78233	1	0.78959	0.12637
Lipoic acid metabolism	28	2	0.18414	0.73486	1	0.78959	0.0017
Citrate cycle (TCA cycle)	20	2	0.21916	0.65924	1	0.78959	0.07907
beta-Alanine metabolism	21	2	0.26731	0.57298	1	0.78959	0.05597
Lysine degradation	30	1	0.29053	0.53681	1	0.78959	0
Biotin metabolism	10	1	0.29053	0.53681	1	0.78959	0
Glyoxylate and dicarboxylate metabolism	32	5	0.30569	0.51472	1	0.78959	0.10582
Arginine and proline metabolism	36	3	0.35256	0.45276	1	0.78959	0.02442
Selenocompound metabolism	20	1	0.39425	0.40423	1	0.78959	0
Glycine, serine and threonine metabolism	34	4	401	0.39686	1	0.78959	0.28464
Histidine metabolism	16	3	0.4334	0.36311	1	0.78959	0.13934
Alanine, aspartate and glutamate metabolism	28	5	0.46408	0.33341	1	0.78959	0.3109
Purine metabolism	71	3	0.53939	0.2681	1	0.78959	0.06162
Glycerophospholipid metabolism	36	1	0.54762	0.26152	1	0.78959	0.02582
Taurine and hypotaurine metabolism	8	1	0.5764	0.23927	1	0.78959	0.42857
Valine, leucine and isoleucine degradation	40	1	0.58148	0.23547	1	0.78959	0
Valine, leucine and isoleucine biosynthesis	8	1	0.58148	0.23547	1	0.78959	0
Pantothenate and CoA biosynthesis	20	1	0.58148	0.23547	1	0.78959	0
Pyrimidine metabolism	39	1	0.59219	0.22754	1	0.78959	0
Primary bile acid biosynthesis	46	2	0.64303	0.19177	1	0.82308	0.04478
Glutathione metabolism	28	3	0.7106	0.14837	1	0.87459	0.36435
Arginine biosynthesis	14	2	0.78466	0.10532	1	0.89676	0.11675
Nitrogen metabolism	6	2	0.78466	0.10532	1	0.89676	0
Porphyrin metabolism	31	2	0.81828	0.087099	1	0.90293	0
Propanoate metabolism	22	1	0.85001	0.070576	1	0.90668	0
Nicotinate and nicotinamide metabolism	15	1	0.94851	0.022956	1	0.97376	0.1943
Butanoate metabolism	15	2	0.97376	0.011548	1	0.97376	0
